# Microstructure and Mechanical Properties of Aluminum Alloy Substrate Material Using Wire-Laser Directed Energy Deposition Assisted with Liquid Nitrogen Cooling

**DOI:** 10.3390/ma19142965

**Published:** 2026-07-09

**Authors:** Fawu Xiang, Ruihao Zhang, Tingqing Cheng, Likun Yang, Hui Gao, Yingying Huang, Haihe Jiang, Jiangang Wang

**Affiliations:** 1 Science Island Branch, Graduate School of USTC, Hefei 230026, China; 2 Anhui Province Key Laboratory of Medical Physics and Technology, Institute of Health and Medical Technology, Hefei Institutes of Physical Science, Chinese Academy of Sciences, Hefei 230031, China; 3 HGTECH Co., Ltd., Wuhan 430223, China; 4 HG Laser Engineering Co., Ltd., Wuhan 430075, China

**Keywords:** coaxial wire-laser system, additive manufacturing, aluminum alloy, liquid nitrogen cooling

## Abstract

Heat accumulation during wire-laser directed energy deposition (WL-DED) may cause the thermal softening of thin aluminum alloy substrates. In this study, a liquid nitrogen-assisted cooling platform was introduced to regulate the substrate temperature during WL-DED of a 6061 aluminum alloy substrate with 5356 aluminum alloy wire. The results show that substrate cooling can mitigate substrate softening, and −100 °C provides improved substrate-bottom hardness while maintaining acceptable bonding quality. The hardness variation is discussed in relation to reduced thermal exposure, grain-size variation, recrystallization behavior, and the possible retention of strengthening phases. This work establishes a preliminary basis for tailoring the local properties of thin aluminum alloy substrates in WL-DED. Since the substrate is not removed, but forms an integrated component of the final assembly along with the deposited material, its properties are critical to component performance. This integrated approach also enhances material utilization and streamlines production by eliminating substrate separation steps.

## 1. Introduction

As electronic products become increasingly lighter and thinner, along with the growing demand for higher production efficiency, greater requirements are imposed on material properties and processing technologies [1,2]. Due to its excellent physical and mechanical properties [3,4]—such as high specific strength, outstanding corrosion resistance, and good ductility—aluminum alloys have been widely used in key industries including electronics, automotive, aerospace, marine, and biomedical sectors [5,6]. AM (advanced manufacturing) is an advanced manufacturing technology that directly forms digital models into three-dimensional solid parts through a “discrete-stacking” layer-by-layer material addition approach. This technology offers advantages such as a wide range of substrate materials, the easy control of feedstock, and programmable cladding geometries [7,8,9]. It enables the manufacture of complex components with benefits including a small heat-affected zone, high precision, controllable processes, and support for multi-directional deposition. The fabricated components exhibit good quality, low residual stress, and minimal distortion [10,11,12].

The current research focus of laser additive manufacturing (LAM) is mainly on the performance of the cladding deposited layer. For example, Le Wan et al. [13] used an aluminum alloy substrate with a thickness of 6.0 mm for thermal field-assisted ring laser directed energy deposition and subsequently investigated the dimensional accuracy and stability of the additively manufactured thin-walled component, achieving a surface roughness of 2.3 μm with uniform microstructure and elemental distribution. Yu Mao et al. [14] used a 2219 aluminum alloy substrate with a thickness of 5 mm to fabricate Al-Cu-Sc alloy via beam oscillating wire-feed laser directed energy deposition and found that oscillation-assisted additive manufacturing can refine the grain structure and improve mechanical properties. The substrate thicknesses used in the above experiments are generally large (all greater than 5 mm), and there is a lack of systematic research on how the LAM process affects the substrate itself when thinner substrates are used in practical applications. Given the increasing demand for the combined use of the substrate and the deposited layer in electronic products (e.g., laptops, mobile phones, and watches), there is an urgent need to conduct systematic research on substrate thinning and its impact on the mechanical properties of the substrate.

During laser manufacturing processes [15,16], the input of laser energy raises the temperature of the substrate material, leading to a decrease in material hardness [17]. This phenomenon is particularly pronounced in laser welding. Multiple experimental studies on aluminum alloys have confirmed this issue. For example, Jie Sheng et al. [18] found in their welding experiments on AA6061-T6 aluminum alloy that the substrate hardness was 100 HV, while the hardness in the fusion zone and heat-affected zone dropped to 60–70 HV. D. Narsimhachary et al. [19] investigated the microstructure and mechanical properties of laser-welded 6061 aluminum alloy and measured a reduction in microhardness of approximately 50% in the weld zone compared to the substrate. In practical laser processing applications, increasing the hardness of the substrate is a critical issue that urgently needs to be addressed [20,21,22,23].

As originally demonstrated by Hall and Petch through empirical work, the initial yield stress σ_y_ of low-carbon steels varies with the grain size D as described by the now-standard Hall–Petch relationship [24], which takes the form of Equation (1):

(1) $$\sigma_{y}=\sigma_{0}+k\frac{1}{\sqrt{D}}$$

Here, σ_0_ and k are constants whose values are dictated by the chemistry and microstructure, respectively. This equation shows a strong correlation between the mechanical properties of a material and its grain size.

For aluminum alloys, the main processing challenges are hot cracking and elemental micro-segregation, rather than residual stress. Preheating, commonly used for steels or titanium alloys to reduce thermal shock, tends to prolong the mushy zone lifetime in Al alloys, increasing the risk of solidification cracking. In contrast, a cold platform accelerates heat extraction and creates a steeper thermal gradient ahead of the solid–liquid interface, offering two key benefits: (1) the reduced segregation of alloying elements (Mg, Cu) that form low-melting-point intermetallic phases, and (2) a finer dendritic or equiaxed microstructure that enhances mechanical properties. Additionally, the cold platform mitigates cumulative heat effects in multilayer deposition, preventing excessive thermal cycling that could cause precipitate coarsening in remelted zones.

Existing research has demonstrated that the introduction of liquid nitrogen cooling can effectively control material temperature, thereby improving mechanical properties such as hardness. S. Ajithkumar et al. [25] conducted a study on the microstructure and properties of Inconel 686 alloy fabricated by gas metal arc wire directed energy deposition and found that cryogenic treatment with liquid nitrogen enhanced the mechanical properties of the alloy. Alexey Kuprienko et al. [26] investigated the effect of liquid nitrogen cryogenic cooling during arc directed energy deposition of nickel–aluminum–bronze alloy, and the results showed that this approach refines the grain structure while improving the mechanical properties of the alloy and reduces the distortion. Based on the above relevant literature, it is evident that rapid cryogenic cooling enables fine grain sizes, thereby enhancing the mechanical properties of materials [27,28]. Y. T. Chang et al. [29] used the rapid cooling and thermal cycling inherent to laser DED to produce 304L stainless steel with a bimodal grain size and cellular dislocations. Their work showed that, regardless of the print strategy, DED-built 304L stainless steel had better strength and ductility (from room temperature to 77 K) than conventional 304L stainless steel. This suggests that DED is a promising method for producing high-performance stainless steel for cryogenic use. Using a liquid nitrogen cryogenic cooling mode in the WA-DED (wire arc directed energy deposition) of a magnesium alloy, X. Li et al. [30] reported that, relative to air cooling, the cryogenic condition boosted yield strength by 50%, tensile strength by 58%, and elongation by 174%. These improvements resulted from lower porosity, extensive nano-sized precipitates, and grain refinement. These findings clearly demonstrate that cryogenic cooling significantly benefits the microstructure and mechanical properties of additively manufactured metals.

However, all the above studies focus on the performance of the additively deposited materials themselves. Few studies have employed cryogenic treatment to investigate substrate properties (e.g., back-side roughness, microhardness, or microstructural evolution) during or after additive manufacturing, largely because the substrate is conventionally regarded as a sacrificial material that is removed. However, in applications where the substrate is retained as part of the final component—as in the WL-DED process used here—its properties become critical. This gap motivates the present work, which systematically examines the effect of stepwise cryogenic substrate cooling on aluminum alloy substrates during WL-DED.

This paper demonstrates the innovative application of a liquid nitrogen cooling system for the rapid cooling of the aluminum alloy substrate during the WL-DED process. The effect of the cooling temperature gradient on the substrate material was investigated, with a focus on analyzing the microhardness and microstructure at the bottom region of the substrate, which enables the localized tailoring of mechanical properties. This approach offers a practical solution by allowing substrate materials to be directly used in additively manufactured product components, which means that the substrate materials, as a substratum, form an integrated component of the final assembly along with the deposited material.

## 2. Materials, Systems and Experimental Methods

### 2.1. Materials

In this study, a 6061 aluminum alloy plate measuring 100 mm × 50 mm × 3 mm (length × width × thickness) was utilized as the substrate. It offers good mechanical properties and low cost and is frequently used in electronic structural components such as mobile phone frames [31]. A 5356 aluminum alloy wire with a diameter of 1.6 mm was used as the additive manufacturing feedstock material, which possesses excellent mechanical properties and a high commercial utilization rate, making it widely adopted in additive manufacturing, In addition, the use of 5356 wire on a 6061 substrate is a common filler/substrate combination in aluminum alloy joining and repair [32]. Both the substrate and the wire were supplied by TRIO METAL (GZ) Co., Ltd. (TMC, Zengcheng, Guangzhou, China). The chemical compositions (wt.%) were determined using a Thermo Niton XL2 analyzer (Thermo Fisher Scientific, Waltham, MA, USA), and the results are presented in Table 1. Before the deposition process, the substrate surface was cleaned with acetone to remove any oil or grease residues.

### 2.2. Systems

#### 2.2.1. Liquid Nitrogen Cooling System

**Figure 1:**
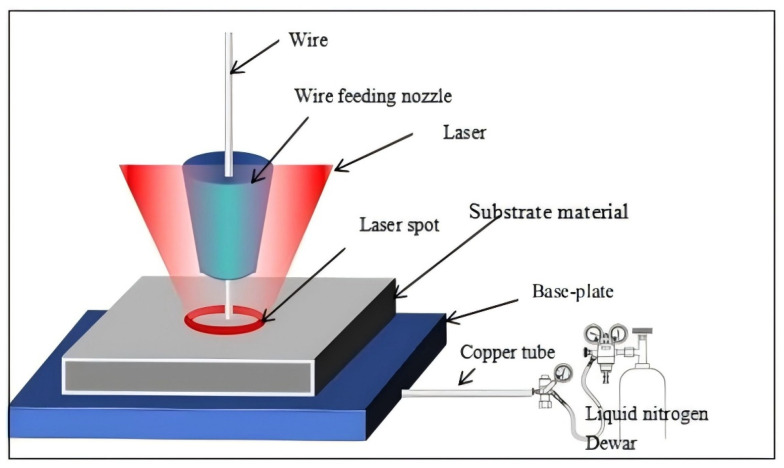
Schematic diagram of the liquid nitrogen cooling and WL-DED platform.

This platform integrated a liquid nitrogen cooling system with precise and controllable flow into the WL-DED technology to regulate the temperature of the substrate material. The overall configuration is shown in Figure 1. Liquid nitrogen was introduced through a copper tube into a sealed copper cavity base-plate, which was in physical contact with the substrate to extract heat from it. A temperature sensor was installed inside the base-plate cavity to monitor the base-plate temperature in real time, and the liquid nitrogen flow rate was controlled at 8–12 L/min. Since the theoretical temperature of liquid nitrogen is −196 °C, in order to rapidly regulate the base-plate cavity temperature, mesh-type resistance heating wires were arranged inside the base-plate. An electric current was passed through these wires to heat them, thereby stabilizing the base-plate temperature within a certain range of the set value. This allows the base-plate to operate within a temperature range of −180 °C to 600 °C.

#### 2.2.2. WL-DED System

The overall configuration of the WL-DED system mainly includes the laser system, the X-Y-Z axis motion control system, a charge-coupled device (CCD) visual positioning system, and a laser processing head integrated with a wire feeding mechanism. The laser (RFL-C6600S, Raycus, Wuhan, China) has a maximum output power of 6000 W and a fiber core diameter of 600 μm, with a wavelength of 1080 ± 5 nm; it operates in a multi-mode beam (M^2^ factor ≤ 12) and features a power stability of ±1.5%. The automatic wire-feed additive manufacturing process proceeds as follows. The X-Y-Z axis motion control system moves the liquid nitrogen cooling system carrying the substrate to the processing position. The CCD vision positioning system then performs visual positioning of the substrate. Subsequently, the laser head activates argon shielding gas and wire feeding. Once the wire contacts the substrate, laser output is triggered to melt the wire and the substrate surface. The system moves along a preset trajectory (e.g., straight line, circle, or spiral). After the trajectory is completed, the wire feeding mechanism retracts, and the laser is turned off with a delay to allow the residual heat to melt and break the wire, thus completing the WL-DED process.

### 2.3. Methods

#### 2.3.1. WL-DED Process Experiments

**Figure 2:**
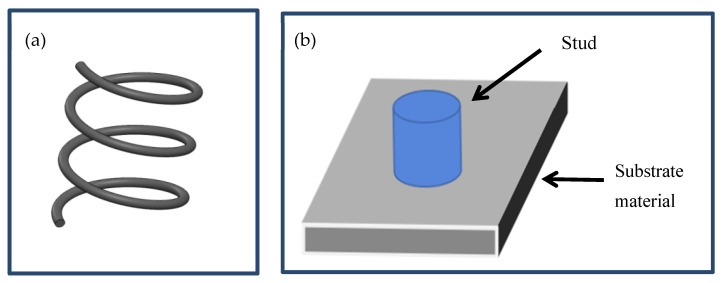
(**a**) Circular continuous ascending path (**b**) Schematic diagram of stud additive manufacturing.

A continuously rising circular trajectory (Figure 2a) was adopted for additive manufacturing. The constant process parameters included a laser power of 4200 W, a wire feeding speed of 2 m/min, a travel speed of 10 mm/s, and an argon flow rate of 10 L/min. The laser conditions, initially derived from Fawu Xiang et al. [33], were further validated through extensive experiments to confirm their suitability for the present study. The final parameter set was chosen because it produced a stable molten pool, continuous deposition, acceptable bead morphology, and no visible cracks or pores under the present circular deposition path. During this process, the wire was delivered through the nozzle. Upon contact with the substrate, the wire closed an electrical circuit, which then triggered the laser signal. This procedure ensured consistency in the initial amount of wire fed each time and stabilized the additive manufacturing process. A stud structure was manufactured on a 6061 aluminum alloy substrate, as shown in Figure 2b, where the gray area represents the substrate and the blue area represents the manufactured zone—the stud.

#### 2.3.2. Optical and Mechanical Properties Measurement

The morphology and microstructure of the WL-DED formed specimen were tested and characterized. The specimen was cross-sectionally cut using a cutting machine, then ground with silicon carbide abrasive papers of 600, 800, 1000, and 2000 grit sizes and etched with Keller’s reagent (2.5 mL HNO_3_, 1.5 mL HCl, 1 mL HF dissolved in 95 mL H_2_O) for 60 s. The macroscopic morphology of the additively manufactured specimen was examined using an optical microscope (KEYENCE VHX-600, Osaka, Japan).

The ground specimen was mechanically polished and then subjected to anodic film coating. The specific procedure was as follows: the prepared specimen served as the anode, and a stainless-steel plate served as the cathode. The polished surface of the specimen was immersed in the electrolyte (20 mL of 40 vol% fluoroboric acid diluted with distilled water to 1000 mL). The process parameters were a voltage of 20–30 V applied for 1–3 min. Immediately after the film coating was completed, the specimen surface was rinsed with anhydrous ethanol to remove residual electrolyte and then dried with cold air for later use. The metallographic structure of the additively manufactured specimen was examined using a metallurgical microscope (Leica DM4000M, Wetzlar, Germany).

**Figure 3:**
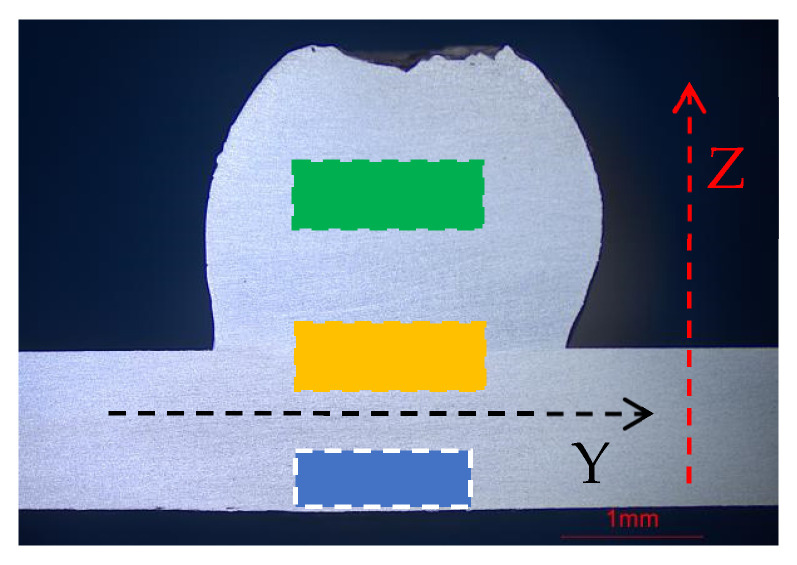
Diagram of the microhardness testing area.

Microhardness testing was performed using a Vickers microhardness tester (HV-1000SPTA, Laizhou, China). A test load of 300 gf was selected, with a loading time of 15 s. The study focused on the substrate bottom, the fusion zone, and the AMZ (additive manufacturing zone), as shown in Figure 3, where the blue (substrate bottom), yellow (fusion zone), and green areas (AMZ) represent these three regions, respectively. Test points were taken at intervals of 0.5 mm in the longitudinal direction (Z-direction, indicated by the red dashed arrow) from the substrate bottom to the AMZ. Additionally, at each longitudinal position, three test points were taken at intervals of 0.5 mm in the transverse direction (Y-direction, indicated by the black arrow). The average microhardness value of these three points was used as the microhardness value at that longitudinal position—that is, the microhardness at that position was represented by the average of three measurements. The measurement accuracy of the microhardness tester is ±3% HV according to the manufacturer’s calibration certificate.

The material structure and chemical composition were characterized using a scanning electron microscope (SEM) (GeminiSEM 360, Carl Zeiss, Oberkochen, Germany) with an elemental analyzer (EDS).

Electron backscatter diffraction (EBSD) (model: DAX Hikari Plus, FEI Nova NanoSEM 450, Brno, Czech Republic) was used to analyze the grain size, grain orientation distribution, and texture characteristics. The EBSD step size was set to 2 μm, which is less than one-tenth of the average grain size (approximately 20 μm) of the 6061 aluminum alloy, ensuring sufficient data points per grain for reliable orientation analysis.

## 3. Results and Discussion

### 3.1. Parameters Comparing

To qualitatively investigate the effect of progressively decreasing substrate temperature (from 20 °C to −180 °C in 40 °C steps) on the back-side roughness and microhardness of the substrate after WL-DED, a series of experiments was conducted at six different temperatures: 20 °C (room temperature baseline), −20 °C, −60 °C, −100 °C, −140 °C, and −180 °C. For each condition, three independent WL-DED experiments were performed to ensure reliability and repeatability. The process parameters (laser power: 4200 W; wire feed speed: 2 m/min; travel speed: 10 mm/s) were kept constant across all experiments to isolate the effect of cooling temperature.

This was done in preparation for future work that will use a greater number of intermediate temperature points to further clarify the transition behavior. This approach is expected to narrow the range for subsequent precise temperature control, while saving experimental materials and time, and providing a data reference for practical production.

**Figure 4:**
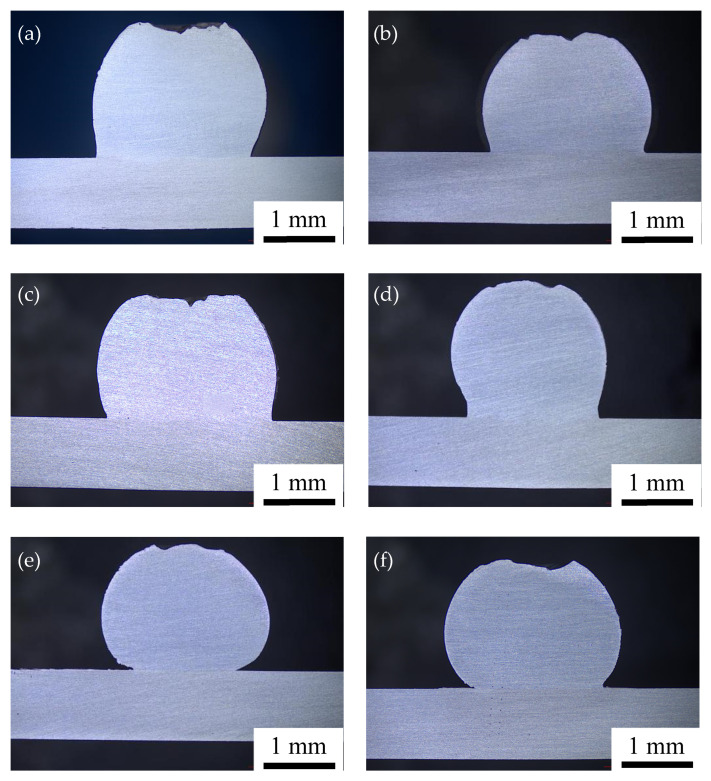
Cross-sectional images of the substrate and AMZ at different temperatures: (**a**) 20 °C, (**b**) −20 °C, (**c**) −60 °C, (**d**) −100 °C, (**e**) −140 °C, (**f**) −180 °C.

Figure 4 shows cross-sectional images of aluminum alloy WL-DED specimens obtained under different liquid nitrogen temperature gradients. Figure 4a–f correspond to substrate temperatures of 20 °C, −20 °C, −60 °C, −100 °C, −140 °C, and −180 °C, respectively. No obvious cracks or pores were observed inside the AMZ. In Figure 4a–d, the AMZ and the substrate are well fused. However, Figure 4e shows a noticeable gap at the interface between the substrate and the AMZ, indicating poor fusion. At −140 °C and −180 °C, frost or condensates formed on the substrate surface. Although the laser power was increased from 4200 W to 5000 W in additional trials, sound bonding was still not achieved. This indicates that poor interfacial bonding was not caused only by insufficient laser energy input but was also related to the deterioration of the substrate surface condition and wetting behavior caused by frost/condensates. Therefore, excessive cooling is not suitable for stable WL-DED under the present setup.

The substrate is a critical factor determining the success of the WL-DED process; under normal conditions (20 °C to −100 °C), the substrate surface remains dry, which promotes good spreading and fusion. However, at −140 °C and −180 °C, the presence of water effectively dewets the molten metal. This observation highlights that the substrate surface condition (specifically the absence of condensates) is as important as the temperature itself for ensuring proper wetting.

**Figure 5:**
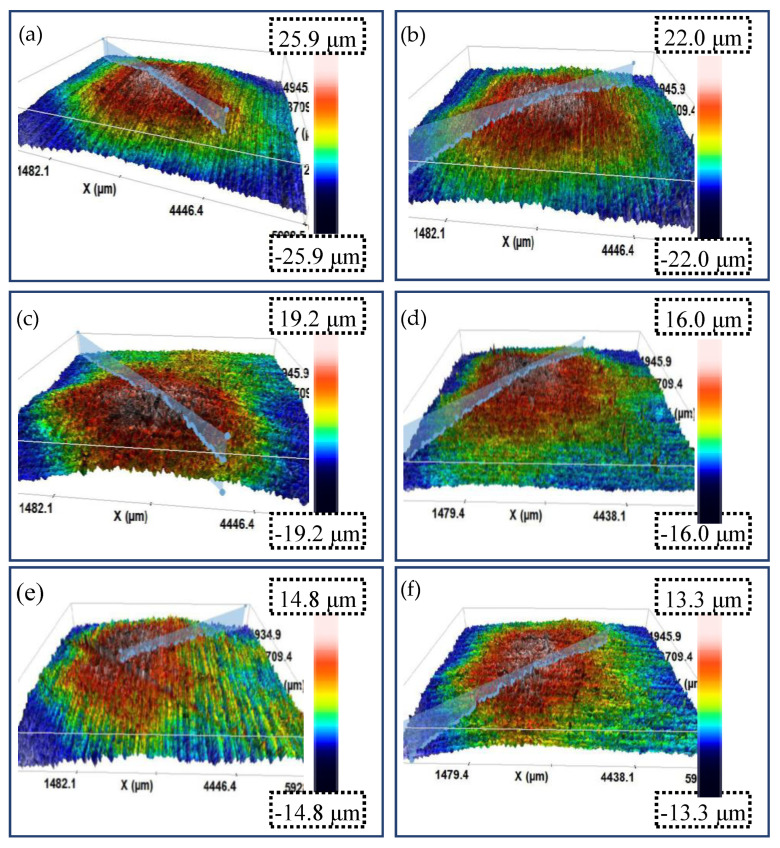
Deformation amount on the back side of the substrate at different temperatures: (**a**) 20 °C, (**b**) −20 °C, (**c**) −60 °C, (**d**) −100 °C, (**e**) −140 °C, (**f**) −180 °C.

Figure 5 shows the deformation amount on the back side of the substrate of aluminum alloy WL-DED specimens obtained under different liquid nitrogen temperature gradients. Figure 5a–f correspond to substrate temperatures of 20 °C, −20 °C, −60 °C, −100 °C, −140 °C, and −180 °C, respectively. It is observed that, as the temperature decreases, the back side deformation amount of the substrate gradually diminishes. This is because the lower the initial temperature of the substrate, the less heat generated during the WL-DED process is transferred to the back side of the substrate, resulting in smaller deformation. The relationship between the back side deformation amount of the substrate and the set temperature is shown in Table 2, The deformation gradually decreases from 43.0 μm at 20 °C to 25.9 mm at −180 °C (Table 2), indicating a clear temperature-dependent trend.

### 3.2. Microhardness Analysis

**Figure 6:**
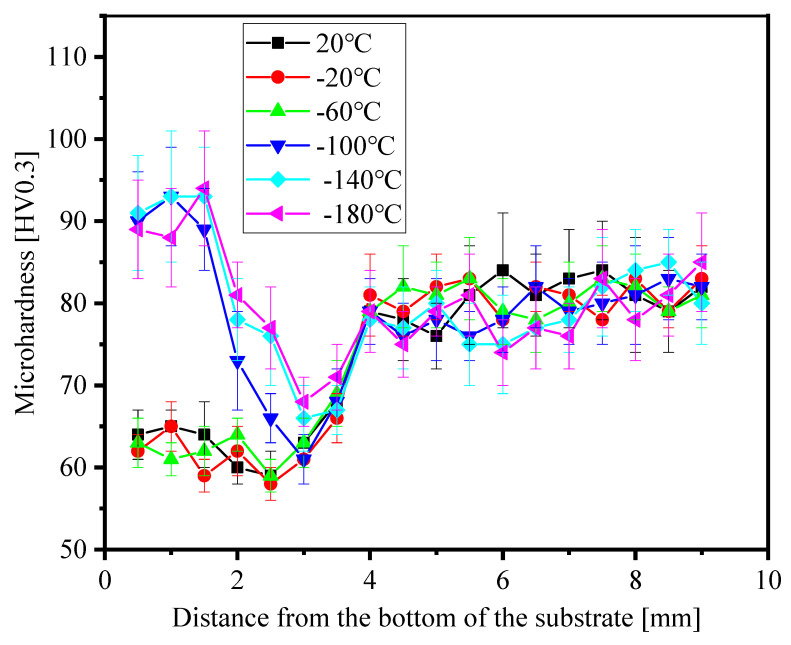
Microhardness of different zones.

Figure 6 presents the microhardness test results of the substrate, fusion zone, and AMZ under different cooling temperatures, and the standard deviations are provided as error bars in Figure 6. The as-received 6061 substrate showed a microhardness of 80–100 HV0.3. After WL-DED, the substrate-bottom hardness decreased to 55~65 HV0.3 at 20 °C, −20 °C, and −60 °C, indicating obvious thermal softening. At −100 °C, the substrate-bottom hardness recovered to 85~95 HV0.3 while sound bonding between the AMZ and the substrate was still maintained. Although relatively high hardness values were also observed at −140 °C and −180 °C, these two conditions caused poor interfacial bonding due to frost/condensate formation. Therefore, −100 °C was identified as the preferred condition within the present processing window.

The microhardness of the deposited track remained largely unchanged across all cooling temperatures, indicating that the solidification microstructure of the additively manufactured zone is robust against substrate temperature variations (given the substrate thickness of 3 mm). Therefore, the cooling temperature does not directly affect the deposited track. This suggests that either the deposited material is less thermally sensitive than the substrate, or that the substrate’s temperature variations are insufficient to alter the deposit’s properties.

### 3.3. Microstructure and Elemental Analysis

To investigate the effect of liquid nitrogen cooling temperature on the microstructure of aluminum alloy fabricated by WL-DED, we clarified in the revised manuscript that 20 °C and −100 °C were selected as representative conditions: 20 °C represents the non-cryogenic condition, whereas −100 °C represents the effective cryogenic cooling condition that improved substrate hardness while maintaining sound interfacial bonding. These two conditions were selected primarily to compare the effects on grain characteristics and mechanical properties.

**Figure 7:**
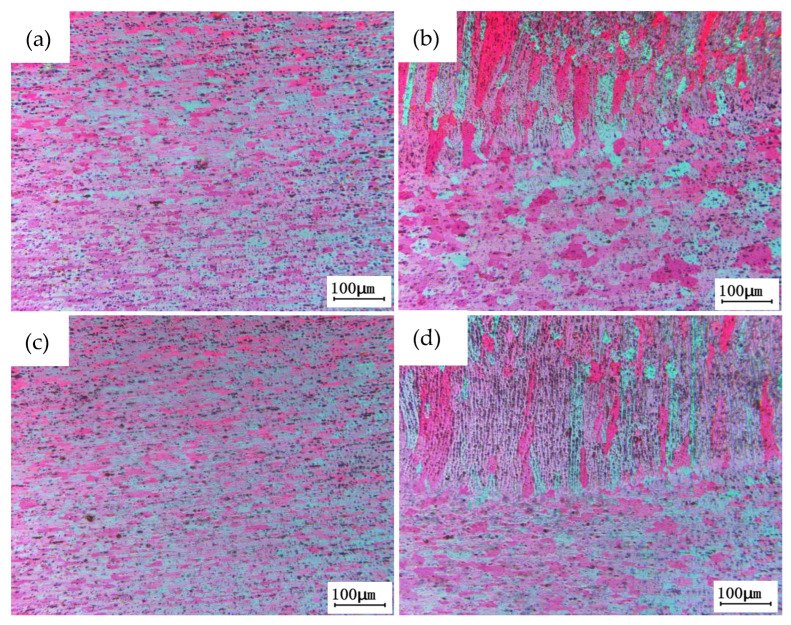
Metallographic structures at different temperatures: (**a**) substrate bottom at 20 °C, (**b**) fusion zone and AMZ at 20 °C, (**c**) substrate bottom at −100 °C, (**d**) fusion zone and AMZ at −100 °C.

The metallographic structures at 20 °C are shown in Figure 7a,b. Figure 7a shows the substrate bottom: the grains exhibit a flattened strip/fiber shape along the rolling direction, which is a typical characteristic of plastic deformation. The grain size is non-uniform, with fine recrystallized grains and large deformed grains coexisting, indicating a partially recrystallized state. In the fusion zone (lower part of Figure 7b), the grains transform into an equiaxed shape with uniform size, and partial recrystallization occurs. In the AMZ (upper part of Figure 7b), the grains are strongly elongated in the vertical direction, forming fibrous columnar grains, indicating that the heat generated by the laser conducts toward the substrate bottom.

The metallographic structures at −100 °C are shown in Figure 7c,d. Figure 7c shows the substrate bottom, where the grains exhibit a flattened strip/fiber shape along the rolling direction with uniform grain size, and both recrystallized and deformed structures are present. In the fusion zone (lower part of Figure 7d), the grains transform into an equiaxed shape with uniform size and clear boundaries, and partial recrystallization occurs. The AMZ (upper part of Figure 7d) is similar to the upper part of Figure 7b, with grains strongly elongated in the vertical direction, forming fibrous columnar grains.

**Figure 8:**
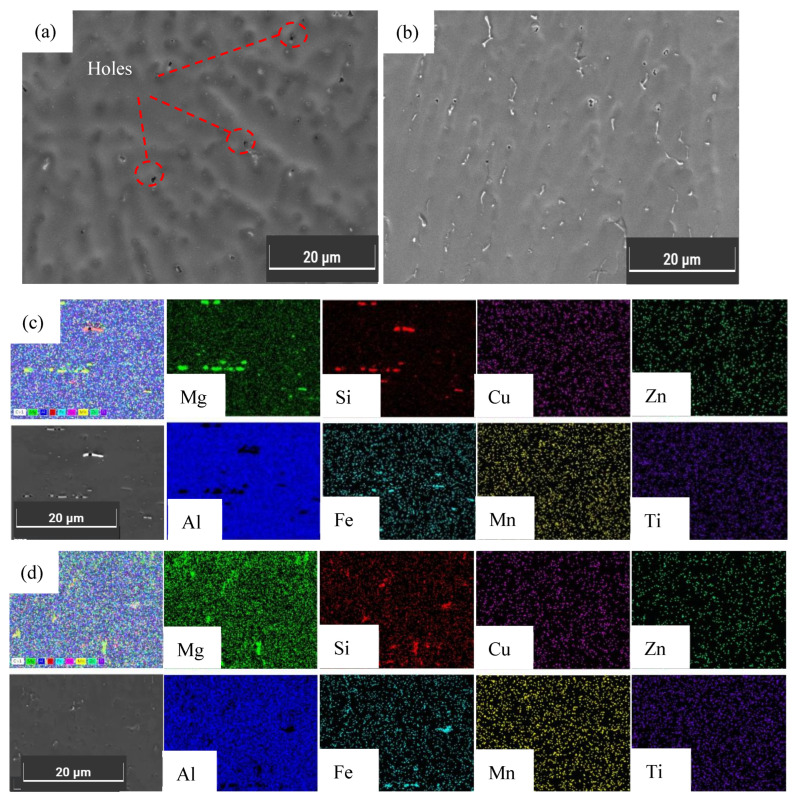
SEM and EDS analysis: (**a**) SEM at 20 °C, (**b**) SEM at −100 °C, (**c**) EDS at 20 °C, (**d**) EDS at −100 °C.

The elemental composition of the substrate bottom was analyzed using SEM and EDS (Figure 8). Figure 8a and Figure 8c show the 2000× magnification image and the EDS mapping of the substrate bottom after WL-DED at 20 °C, respectively. Some fine pores can be observed in Figure 8a. This is because during laser heating, heat is transferred to the substrate bottom, causing the loss of some magnesium elements. The EDS results (Figure 8c) show that Al is the main element, with Mg and Si present. The dark areas represent the aluminum matrix, while the bright areas correspond to secondary phases. Mg is uniformly distributed, whereas Fe exhibits an island-like distribution that is complementary to the distribution of the Al element.

Table 3 shows the elemental composition of this region, with an Mg-to-Si atomic ratio of approximately 2:1. The nominal composition of the 6061 alloy is Al–1.34% Mg_2_Si (mass fraction), as reported by M. Cai [34]. Cheun et al. [35] quantitatively characterized the spatial heterogeneity of the Mg_2_Si precipitate phase in the 6061 aluminum alloy and confirmed its presence within the alloy matrix. Reference [36] also reported Mg- and Si-rich particles. Based on these reports and the known nominal composition, we propose the possible presence of Mg_2_Si as a reasonable hypothesis. However, we acknowledge that future work employing TEM or XRD-based phase identification is necessary to quantify the phase contribution.

The Mg_2_Si phase is beneficial for the precipitation strengthening effect [37]. Figure 8b and Figure 8d show the 2000× magnification image and the EDS mapping of the substrate bottom after WL-DED at −100 °C, respectively. As shown in Figure 8b, no obvious pores are observed, suggesting a reduced loss of magnesium elements. The EDS results (Figure 8d) are consistent with those in Figure 8b, and the Mg-to-Si atomic ratio further confirms the presence of strengthening phases such as the Mg_2_Si phase at the substrate bottom [38,39]. Notably, more Mg_2_Si phase exists at −100 °C than at 20 °C, which partly explains the higher microhardness observed at the substrate bottom after processing at −100 °C compared to that at 20 °C.

### 3.4. EBSD Analysis

**Figure 9:**
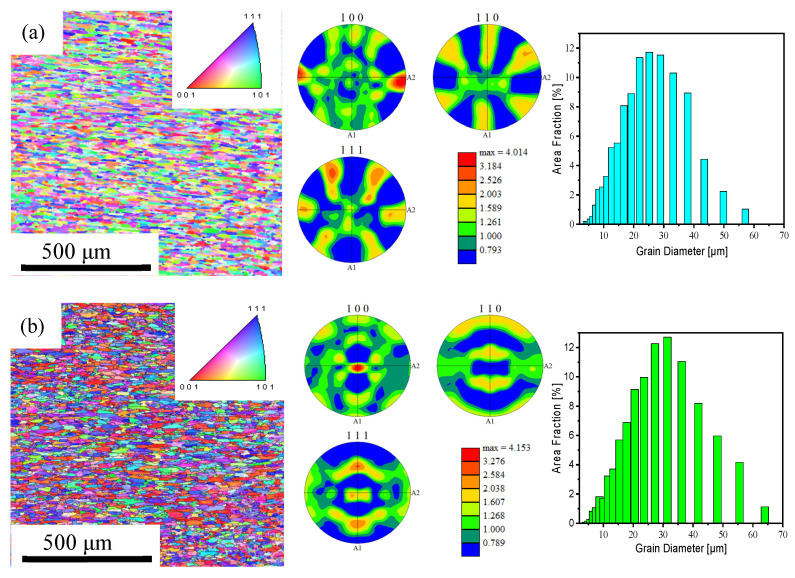
Inverse pole figures of the substrate bottom structure: (**a**) −100 °C; (**b**) 20 °C.

To further investigate the effect of liquid nitrogen cooling temperature on the microstructure of aluminum alloy fabricated via WL-DED, EBSD was used for crystallographic analysis. Figure 9 shows the EBSD analysis results of the substrate regions at 20 °C and −100 °C. Regarding texture intensity, the value at the substrate bottom after WL-DED at 20 °C was 4.01, while that at −100 °C was higher (4.15). Grain size was measured by EBSD and is shown in Table 4. At the substrate bottom after WL-DED at −100 °C, the proportion of grains smaller than 10 μm was 7.5% (compared to 4.2% at 20 °C), and the proportion of grains between 10 μm and 20 μm was 31.1% (compared to 21.2% at 20 °C). The average grain size was 24.7 μm at −100 °C, compared to 28.3 μm at 20 °C. Overall, the proportion of grains with a diameter below 20 μm during WL-DED at −100 °C was more than 50% higher than that during WL-DED at 20 °C. These results indicate that WL-DED at −100 °C effectively refines the grain size at the substrate bottom. Consequently, the higher microhardness observed at −100 °C could be partially explained by the finer grain size achieved at the lower temperature via the fine-grain strengthening mechanism [40,41].

**Figure 10:**
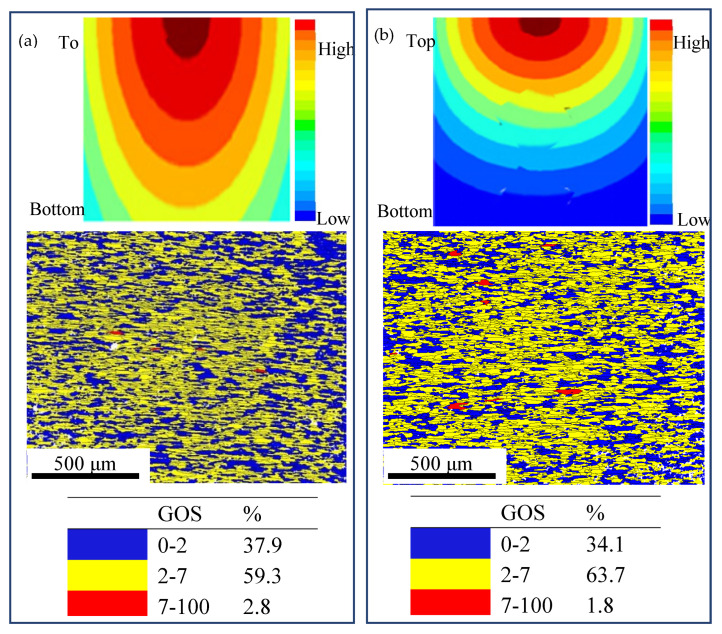
Schematic diagram of substrate temperature conduction and GOS: (**a**) 20 °C, (**b**) −100 °C.

Figure 10a shows the temperature conduction schematic and grain orientation spread (GOS) map of the substrate after WL-DED at 20 °C. It can be seen that heat conducted from the top of the substrate to the bottom region, resulting in a relatively high temperature at the substrate bottom. The corresponding EBSD GOS map is presented, where the blue region represents the recrystallized zone (GOS value ≤ 2), the yellow region represents the substructured zone (2 < GOS value ≤ 7), and the red region represents the deformed zone (GOS value > 7) [40,42]. The map shows that the recrystallized zone accounts for 37.9%, the substructured zone accounts for 59.3%, and no deformed grains are present.

Figure 10b shows the temperature conduction schematic and the GOS map of the substrate after WL-DED at −100 °C. It can be observed that the high-temperature region existed only at the top of the substrate. Due to the presence of liquid nitrogen cooling at the substrate bottom (temperature of −100 °C), the heat was promptly conducted away, resulting in a relatively low temperature in the bottom region of the substrate. The EBSD GOS map shows that recrystallized grains account for 34.1%, substructured grains for 63.7%, and no deformed grains are present. Notably, the proportion of the recrystallized zone at the substrate bottom after WL-DED at −100 °C is 3.8 percentage points lower than that at 20 °C. This difference is primarily attributed to the different cooling temperatures used in the two processes. In the −100 °C process, laser-generated heat is promptly conducted away, keeping the substrate bottom at a low temperature and preventing grain growth. In contrast, after WL-DED at 20 °C, the substrate bottom receives heat from the laser, leading to a higher temperature there and promoting the formation of a coarser grain structure [43]. The greater heat input promotes an increased proportion of recrystallized zones and grain growth, resulting in a decrease in microhardness [44].

The present work should be regarded as a proof-of-concept study demonstrating that liquid nitrogen-assisted platform cooling can reduce substrate softening during WL-DED. Further tensile testing, fatigue testing, residual stress measurement, and component-level validation are required to evaluate practical engineering performance.

The study by Asgharzadeh et al. [45] established a comprehensive strength–hardness empirical relationship applicable to all aluminum alloys by collecting datasets from 16 independent studies. It then experimentally validated this relationship using heat-treated AA6061 and AA7075 tubular specimens. The study found a good linear relationship between tensile strength and hardness, while a binomial relationship existed between yield strength and hardness. Furthermore, the reliability of the mechanical property prediction model based on hardness measurements was verified through finite element analysis, indicating that hardness measurement is an effective alternative method for evaluating the mechanical properties of aluminum alloys. Based on the above findings, the increase in substrate microhardness achieved at a cooling temperature of −100 °C is beneficial for enhancing the reliability of mechanical properties.

## 4. Conclusions

In this study, WL-DED with rapid liquid nitrogen cooling of the substrate was used to systematically investigate the effect of cooling temperature on the additive manufacturing of 6061 aluminum alloy. Through microhardness analysis, microstructural characterization, EDS energy spectrum analysis, and EBSD crystallographic analysis, the following main conclusions were drawn:

(1) At 20 °C, −20 °C, and −60 °C, the cooling effect was insufficient to prevent substrate softening. At −140 °C and −180 °C, frost/condensate formation caused poor interfacial bonding, although the substrate-bottom hardness was relatively high. Therefore, −100 °C was selected as the preferred condition within the present experimental window because it improved substrate hardness while maintaining sound bonding between the AMZ and the substrate. Microstructural analysis revealed that at −100 °C, the average grain size at the substrate bottom was 24.7 μm, which was finer than that at 20 °C (28.3 μm), indicating a grain refinement effect. This becomes one of the possible factors that enhance microhardness.
(2) EBSD analysis further revealed the effect of cooling temperature on recrystallization. At −100 °C, liquid nitrogen cooling promptly dissipated heat, inhibiting grain growth and the recrystallization process. This becomes another potential factor contributing to the increase in microhardness.
(3) Liquid nitrogen cooling-assisted WL-DED of aluminum alloy leads to superior microhardness performance. This enables the localized tailoring of the mechanical properties of WL-DED structural components. This technology has enormous potential for future applications—for example, in the manufacturing of nuts on laptop casings—offering a novel, efficient, and low-consumption solution for the high-quality manufacturing of compact electronic products.

Consequently, such a technique shows promise for applications in the joining processes of micro-devices, including mobile phones, sensors, detectors, and MEMS devices, particularly where hardness, wear resistance, and stress/strain control are critical. It is also effective in mitigating thermal distortion, warpage, and thin-wall deformation, which makes it potentially suitable for high-precision manufacturing scenarios. In the manufacturing of integrated composite structures, the deposited zone is required to exhibit high plasticity and impact resistance, while the substrate itself needs high hardness and wear resistance, because both are used together as an integrated component and are subjected to different loading conditions during service. For the on-site laser cladding repair of cracks, corrosion damage, and defects in aircraft aluminum shells and pipelines, the reverse cold gradient constrains the thermal diffusion of the molten pool.

However, the present work is still a proof-of-concept study based mainly on microhardness and microstructural characterization. Further tensile testing, fatigue testing, residual stress analysis, in situ temperature measurement, and component-level validation are required before practical engineering application. Future work will focus on establishing the relationship between platform temperature, actual substrate thermal history, cooling rate, microstructure, and mechanical response.

Future work will also focus on extending the current approach to other alloy systems, exploring the effect of different cryogenic cooling rates on microstructural evolution, and developing a predictive model for thermal gradient control under various processing conditions. In situ monitoring techniques combined with feedback control will also be investigated to further stabilize the repair quality.

## Figures and Tables

**Table 1:** Chemical composition of 6061 and 5356 aluminum alloy [wt. %].

Materials	Si	Fe	Cu	Mn	Mg	Cr	Zn	Ti	Al
6061 aluminum alloy	0.4	0.5	0.3	0.1	1.2	0.18	0.2	0.2	Bal.
5356 aluminum alloy	0.2	0.4	0.1	0.05	5.0	0.1	0.1	0.2	Bal.

**Table 2:** The relationship between temperature and deformation.

Temperature	20 °C	−20 °C	−60 °C	−100 °C	−140 °C	−180 °C
Deformation of Substrate/μm	43.0	33.8	28.0	27.2	26.3	25.9

**Table 3:** Chemical composition of substrate material [wt. %].

Material	Al	Mg	Si	Fe	Cu	Mn
The substrate bottom after processing at 20 °C	96.09	3.24	0.32	0.13	0.12	trace
The substrate bottom after processing at −100 °C	95.73	3.60	0.33	0.14	0.04	trace

**Table 4:** Grain size measured by EBSD [μm].

Grain Size	≤10	>10 to ≤20	>20 to ≤30	>30 to ≤40	>40	Average Values
The substrate bottom after processing at 20 °C	4.2%	21.2%	31.3%	23.7%	19.6%	28.3
The substrate bottom after processing at −100 °C	7.5%	31.1%	34.6%	19.2%	7.6%	24.7

## Data Availability

The original contributions presented in this study are included in the article. Further inquiries can be directed to the corresponding authors.

## Nomenclature

WL-DED	Wire-laser directed energy deposition
AM	Advanced manufacturing
LAM	Laser additive manufacturing
LNCC	Liquid nitrogen cryogenic cooling
CCD	Charge-coupled device
AMZ	Additive manufacturing zone
SEM	Scanning electron microscope
EDS	Elemental analyzer
EBSD	Electron backscatter diffraction
GOS	Grain orientation spread
